# Supplementary material for: The Arabidopsis stomatal polarity protein BASL mediates distinct processes before and after cell division to coordinate cell size and fate asymmetries
Source: Development. 2021 Sep 26;148(18):dev199919. doi: 10.1242/dev.199919 (PMC8512303; doi:10.1242/dev.199919)
Supplement: Supplementary information [file develop-148-199919-s1.pdf]

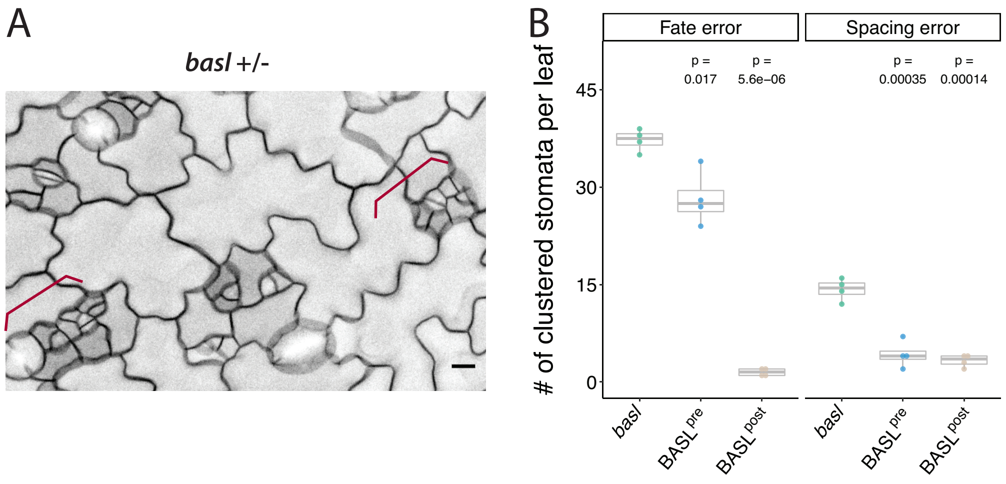

Fig. S1. The epidermal phenotype of *basl* +/- plants and quantification of absolute numbers of stomatal clusters in BASL variant lines.

(A) Confocal image of 5 dpg *BASL* +/- abaxial cotyledon epidermis. Cell outlines are visualized by pATML1::RCI2A-mCherry. Braces indicate clustered small cells. This and similar images are the source data quantified in Figure 1 K-L. Scale bar, 10 µm. (B) Counts of stomatal clusters from which Figure 3D was calculated. P-values are calculated by Student's *t*-test due to small sample sizes. Pairwise p-values comparing individual genotypes to *basl* are presented.

Development • Supplementary information

Table S1. Primers used in this study

Primer name	Sequence (5’-3’)
Cloning of BASL CDS into pENTR R2-BASL Stop-L3	
attB2r-BASL F	GTACAAAGTGGCTATGGCTTCACAGTGG
attB3-BASL Stop R	ATAATAAAGTTGTCAGAATCTACAAC
attB2r_adapter	GGGGACAACTTTGTATAATAAAGTTG
attB3_adapter	CTCTATCACTCACTAAAGAG
Cloning of Venus-DBox into pENTR L1-Venus-DBox-L2	
DBox part1 BamHI cacc F	CTCAACAATCCACGGATGATGTTGTTGTGG
DBox part2 F	CAGGTTTTTGAGATTATCCTCC
DBox part2 R	AAAAAACCTGTAGTCAAGAGAAACGC
DBox part3 F	CTTCTCTCGAGCAGCAACTAAACC
DBox part3 R	GCUGGUGGATCCATGGTGAGCAAGGGCG
BamHI-Venus F	ACCAGCGGATCCCTTGTACAGCTCGTCCATGCC
BamHI-Venus w/o Stop R	GTACAAAGTGGCTGGTACCATGGTGAGCAAGGG
Cloning of BASL promoter into pENTR L4-pBASL-R1	
pBASL F	CCGCGGCCGCGGATATTCATCTTTGCTACACAAGAGTC
pBASL R	CGCGGCCGCGGCTGTTATGTTTGTGTTTCTTTGTCAC
Cloning of KNOLLE promoter into pENTR L4-pKNOLLE-R1	
pKNOLLE F	TGATGATTACCACAAGCTCC
pKNOLLE R	CTTTTTCACCTGAAAGTCAAC
Direct mutagenesis of DBox into mDBox	
Direct mut DBox F	GAAACGGTCAAGTTGTTGGTGA
Direct mut DBox R	TCACCAACAACTTGACCGTTTC

Development • Supplementary information
