# Supplementary material for: The Arabidopsis stomatal polarity protein BASL mediates distinct processes before and after cell division to coordinate cell size and fate asymmetries
Source: Development. 2021 Sep 26;148(18):dev199919. doi: 10.1242/dev.199919 (PMC8512303; doi:10.1242/dev.199919)
Supplement: Reviewer comments [file develop-148-199919_review_history.pdf]

The *Arabidopsis* stomatal polarity protein BASL mediates distinct processes before and after cell division to coordinate cell size and fate asymmetries

Yan Gong, Julien Alassimone, Andrew Muroyama, Gabriel Amador, Rachel Varnau, Ao Liu and Dominique C. Bergmann

DOI: 10.1242/dev.199919

Editor: Yka Helariutta

Review timeline

Original submission: 18 June 2021
Editorial decision: 5 August 2021
First revision received: 12 August 2021
Accepted: 18 August 2021

Original submission

First decision letter

MS ID#: DEVELOP/2021/199919

MS TITLE: Arabidopsis stomatal polarity protein BASL mediates distinct processes before and after cell division to coordinate cell size and fate asymmetries

AUTHORS: Yan Gong, Julien Alassimone, Andrew Muroyama, Gabriel Amador, Rachel Varnau, Ao Liu, and Dominique Bergmann

I have now received all the referees reports on the above manuscript, and have reached a decision. The referees' comments are appended below, or you can access them online: please go to BenchPress and click on the 'Manuscripts with Decisions' queue in the Author Area.

The overall evaluation is positive and we would like to publish a revised manuscript in Development, provided that the referees' comments can be satisfactorily addressed. Please attend to all of the reviewers' comments in your revised manuscript and detail them in your point-by-point response. If you do not agree with any of their criticisms or suggestions explain clearly why this is so.

We are aware that you may currently be unable to access the lab to undertake experimental revisions. If it would be helpful, we encourage you to contact us to discuss your revision in greater detail. Please send us a point-by-point response indicating where you are able to address concerns raised (either experimentally or by changes to the text) and where you will not be able to do so within the normal timeframe of a revision. We will then provide further guidance. Please also note that we are happy to extend revision timeframes as necessary.

Reviewer 1

Advance summary and potential significance to field

The manuscript by Gong et al. presents an innovative genetic tools that allow the very meticulous dissection of the role of BASL in the coordination of cell fate and size assymetries in the stomatal

© 2021. Published by The Company of Biologists under the terms of the Creative Commons Attribution License (https://creativecommons.org/licenses/by/4.0/).

lineage. This is a remarkable piece of work that combine quantitative analysis with live imaging and yield profound new insights on the role of polarity determinants:
- polarisation of BASL and daughter cell size can be uncoupled
- cell fate asymmetry and cell size can be uncoupled
- the division rate of daughter cells of ACD are non cell autonomously coupled via peptide signalling in a BASL-dependent manner.

These results reinforce the idea that plants and animals have settled on different modes to orchestrate differential cellular and developmental behavior in development.

Comments for the author

The manuscript by Gong *et al.* presents an innovative genetic tools that allow the very meticulous dissection of the role of BASL in the coordination of cell fate and size asymmetries in the stomatal lineage. This is a remarkable piece of work that combine quantitative analysis with live imaging and yield profound new insights on the role of polarity determinants:

- polarisation of BASL and daughter cell size can be uncoupled cell fate asymmetry and cell size can be uncoupled
- the division rate of daughter cells of ACD are non cell autonomously coupled via peptide signalling in a BASL-dependent manner.

These results reinforce the idea that plants and animals have settled on different modes to orchestrate differential cellular and developmental behaviour in development.
The manuscript is exemplary in its quality and clarity. I only have minor comments:

- on l159 and 259: adding a one sentence conclusion to each of the sections would further fluidify the reading.
- on l193-194: I found that '*was largely rescued*' could give a false impression. The authors could consider to rephrase the sentence to "*In contrast, the stomatal cluster phenotype was only partially rescued by BASLpost (Figure 3C), with residual clustered stomata arising from spacing errors(...)*"

Reviewer 2

Advance summary and potential significance to field

The manuscript by Gong et al. has the features of many publications from the Bergmann lab, in which experiments are elegantly designed, assays are carefully explained, and rationales are well justified. In this story, by engineering the polarity protein BASL to generate two variants that are predominantly expressed in pre-divisional and post-divisional stomatal lineage cells, respectively, the authors provided experimental evidence that pre-division BASL contributed significantly to the physical asymmetry of stomatal ACD, whilst post-division BASL enforces cell-fate commitment. The concepts that have been hypothesized since the discovery of BASL are now established by long-term live-cell imaging in this study that couples the localization of variants with division and differentiation of the stomatal lineage cells. In addition, by characterization of the division behaviors of the two daughter cells, this study discovered the coordination between the division frequencies of sister cells, and the coordination is mediated by the EPF2 peptide ligand and requires BASL as an effector. The research topic and the new findings are a good fit for the audience of Development. The overall reading of the manuscript is quite pleasant. I do not have major concerns but a few minor suggestions for further strengthening the study.

Comments for the author

1. The expression of the transgenic lines used in this study, such as the BASL variants and EPF2 overexpression, were not fully evaluated for phenotype comparison, in particular, both genes have dosage effect in stomatal development.
It would be helpful to present qPCR data.

2. Fig. 1 B-C should also show an image of basl/+ because quantification indicated the presence of a phenotype (K-L).

© 2021. Published by The Company of Biologists under the terms of the Creative Commons Attribution License (https://creativecommons.org/licenses/by/4.0/).

3. Fig. 4D, the diagram on the left does not need to include the division because the quantifications are only about the pre-division nuclear position.

4. Fig. 5B, it would be help if diagrams can be added to explain the events in the quantification.

5. Lines 162-164 need some explanation for why the two stages of cotyledons were used.

Reviewer 3

Advance summary and potential significance to field

The differences between polarity mechanisms and the functions of polarized proteins in plants vs animals is a long standing and interesting question in developmental biology. The similarities and differences in how these two sets of multi-cellular eukaryotes use polarity in development is of substantial interest. In stomata development in Arabidopsis the polarized protein BASL has a key role in orienting the asymmetric cell divisions that give rise to distinct epidermal cell types and contributes to the downstream cell type specification pathways. In an elegant set of experiments and with careful analysis the authors are able to reveal specific roles of BASL pre-asymmetric cell division and post-division. In cells pre-division, BASL is required for orientation of the cell division plane and post-division it is required for distinct daughter cell fate specification, plus has a role in coordination of subsequent daughter cell divisions.
One of the critical findings here is that a key BASL function can be completed when BASL expression occurs just after the asymmetric cell division and, thus, isn't required to be inherited by the daughter cell during the division. This is different from findings in animals systems where the inheritance of the polar protein is required for cell fate activities. Additionally, the uncoupling of cell size from cell ID in the daughter cells is an important result and the notion that cell size is impacting division potential (not fate) of cells in the stomatal lineage is very, very interesting. This work provides important advancements in our understanding of BASL function but, importantly and much beyond that, has important broad implications for regulation of asymmetric cell divisions and cell identity in plant systems. Finally the potential impact of a cell's polarity domains on neighboring cells can serve as a novel conceptual framework upon which to better understand how plants modulate their development in response a changing environment.

Comments for the author

Congratulations to the authors on a very well written, clear, and exciting manuscript. In addition the figures are well laid out, complete, and lovely. I have very few comments to put forth and the manuscript was a pleasure to read.
One thing that perhaps I just missed was the definition of the abbreviation SD in figure 2. I couldn't find the definition in the legend and didn't see it in the associated text either. I believe providing it in the legend would be very helpful as the abbreviation is used in panels C and E.
Extremely minor comments:
- add a period to the end of the sentence on line 58.
- I suggest changing the asterisks in the size of the fields images to x (388*388 vs. 388x388). For me this would be more clear, but perhaps this is just me. :)
- The color in Fig2C that designates the polarized region as defined by POME is very similar the color for the BASL post (its a peachy color). Perhaps the authors could chose a different color for the polarized regions perhaps lavender or something.
- At line 249, the authors indicate that orientation of "division places to preserve or avoid cell contacts" may be a more important aspect of pre-ACD BASL function. It seems to me the word "cell" is a bit too generic here... all the cells have contacts regardless of their identity. Perhaps they would consider revising it to something more specific such as to "avoid stomatal (or guard cell) contact" or "avoid contact between stomata".

© 2021. Published by The Company of Biologists under the terms of the Creative Commons Attribution License (https://creativecommons.org/licenses/by/4.0/).

First revision

Author response to reviewers' comments

We would like to thank the reviewers for truly collegial evaluations of this work, and for their helpful suggestions to improve it. Below are specific responses to each reviewer. In the revision, we also made some formatting adjustments to fit with journal guidelines. Modified sections of the revised text are highlighted to make them easy to find and review, as the line numbers have changed from the original version.

Reviewer 1 Comments for the Author:
Lines 159 and 259: adding a one sentence conclusion to each of the sections would further fluidify the reading.

The reviewer refers to lines that mark the end of the section on BASL variant localization (159) and BASL variant rescue capacity (259). We have added very brief summaries of the data, as these points are revisited in considerable detail in the discussion.

Lines:193-194: I found that 'was largely rescued' could give a false impression. The authors could consider to rephrase the sentence to "In contrast, the stomatal cluster phenotype was only partially rescued by $BASL^{post}$ (Figure 3C), with residual clustered stomata arising from spacing errors(...)".

The rescue is from ~50% clustered stomata in *basl* to <10% clustered stomata upon expression of $BASL^{post}$. In contrast, expression of $BASL^{pre}$ only reduced the percentage of stomatal in clusters to 40%. Considering these numbers, “largely rescued” seemed an accurate description of $BASL^{post}$ rescue efficacy, while “only partially” suggestion seeming more appropriate for describing $BASL^{pre}$. We therefore substituted “largely” by “substantially”.

Reviewer 2 Comments for the Author:
1. The expression of the transgenic lines used in this study, such as the BASL variants and EPF2 overexpression, were not fully evaluated for phenotype comparison, in particular, both genes have dosage effect in stomatal development. It would be helpful to present qPCR data.

We completely agree with the reviewer’s point that accurate interpretations of the rescue capacity of BASL variants and the dependency of EPF2 overexpression -mediated phenotypes on *BASL* presence require us to ensure that expression levels are comparable between genotypes. The specifics of the developmental questions, however, make qPCR a non-ideal choice for ascertaining BASL variant levels. We explain below (and add text to the methods) to explain the challenges and our solutions to the problem.

(1) On the problem of EPF2 overexpression.
The reviewer raises a very critical point that EPF2 has a dose-dependent effect on suppression of stomata. For this very reason, we went through the extra steps of (1) creating lines of 35S:EPF2 in *basl*, and (2) selecting a line that consistently suppressed stomatal production to an intermediate extent, and then (3) crossing the same line to *basl* and to Col and assaying phenotypes in F1 seedlings to ensure that the 35S:EPF2 dose was the same among the genotypes we were comparing.

The fact that in a *basl* background, EPF2 suppressed entry divisions--but not spacing divisions--whereas both entry and spacing divisions were suppressed in *BASL+/-* is another suggestion that loss of *BASL* has a specific effect on the perception/response to EPF2 rather than having an effect on the levels of *EPF2* transcript being produced.

(2) On the problem of comparing dosages of BASL variants for rescue
This is a tricky problem because we are comparing constructs where we have imposed a strong protein-level regulatory element (the destruction box to decrease protein stability) or altered cell phase and cell type expression (BASL promoter vs. KNOLLE promoter).

qRT-PCR would assay RNA levels in a leaf. Because we already know that we have protein-level

© 2021. Published by The Company of Biologists under the terms of the Creative Commons Attribution License (https://creativecommons.org/licenses/by/4.0/).

control on BASL stability, we know qRT-PCR would be missing the key difference between $BASL^{full}$ and $BASL^{pre}$. $BASL^{post}$ is expressed with the KNOLLE promoter that is active in all dividing cells in the leaf, not just asymmetrically dividing stomatal lineage cells. qRT-PCR on these lines would detect higher expression of *BASL* variants relative to lines where variants are driven by the *BASL* promoter, but the expression level in the relevant asymmetrically dividing cell might be lower (and again, the protein level is really the essential parameter).

We do have a way to estimate protein levels in individual cells and this is through quantitative imaging. We compared the brightness of $BASL^{pre}$ reporter to $BASL^{full}$ reporter before division and $BASL^{post}$ reporter to $BASL^{full}$ reporter after division when all the reporters were imaged under the same settings. Knowing that the $BASL^{full}$ variant was sufficient to fully rescue *basl*, we chose $BASL^{pre}$ and $BASL^{post}$ lines that were at least as bright as $BASL^{full}$ (See Figure R1).

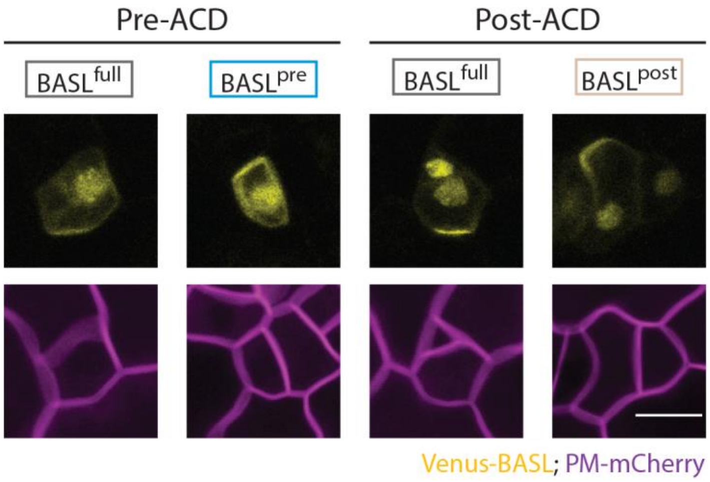

Figure R1. Comparing the brightness of different BASL variant reporters obtained with identical image acquisition settings. Top row (yellow) includes examples of pre-ACD $BASL^{full}$, pre-ACD $BASL^{pre}$, post-ACD $BASL^{full}$, and post-ACD $BASL^{post}$ from the lines used in the paper; reporters exhibit similar brightness. Bottom row (magenta) is a reporter marking cell outlines so that we could chose actively dividing stomatal lineage cells of similar cell sizes for the comparisons. Scale bar 10 µm, all images at same magnification.

In addition, we would argue that $BASL^{pre}$ and $BASL^{post}$ represent qualitatively different manipulations, and they differ in their capacity to rescue discrete phenotypes: (1) stomatal pairs caused by fate or spacing errors and (2) nuclear migration. $BASL^{pre}$ could rescue pre-division nuclear migration better than $BASL^{post}$, whereas $BASL^{post}$ rescued fate errors much better.

We added more explanation of how comparable BASL variant lines were selected in the methods section.

2. Fig. 1 should also show an image of basl/+ because quantification indicated the presence of a phenotype (K-L).

An image of the basl/+ phenotypes was added to Supplemental Figure 1A

3. Fig. 4D, the diagram on the left does not need to include the division because the quantifications are only about the pre-division nuclear position.

We think it is useful to include the division to indicate that nuclear position is being measured before division in cells that were tracked such that we know they would eventually divide asymmetrically.

© 2021. Published by The Company of Biologists under the terms of the Creative Commons Attribution License (https://creativecommons.org/licenses/by/4.0/).

4. Fig. 5B, it would be help if diagrams can be added to explain the events in the quantification.

Cartoons indicating the division types were added to Fig. 5B

5. Lines 162-164 need some explanation for why the two stages of cotyledons were used.

The two phenotypes: clusters of small cells (precursors) and clusters of stomata are most commonly measured at mid-development (4-5 dpg) and mature (14+ dpg) stages respectively (Dong et al., 2009; Rowe et al., 2019; Simmons et al., 2019). We added a brief note in the text to explain our choice of time points.

Reviewer 3 Comments for the Author:

One thing that perhaps I just missed was the definition of the abbreviation SD in figure 2. I couldn't find the definition in the legend and didn't see it in the associated text either. I believe providing it in the legend would be very helpful as the abbreviation is used in panels C and E.

SD is standard deviation, and it is shorthand for the way the outputs of POME analysis represent polar crescent size. We have now defined this in the legend and added a further explanatory note in the text.

Extremely minor comments:
- add a period to the end of the sentence on line 58.

Done!

- I suggest changing the asterisks in the size of the fields images to x (388*388 vs. 388x388). For me this would be more clear, but perhaps this is just me. :)

We changed * to x throughout

- The color in Fig2C that designates the polarized region as defined by POME is very similar the color for the BASL post (its a peachy color). Perhaps the authors could chose a different color for the polarized regions, perhaps lavender or something.

We changed this to a lavender-ish color and gave the BASL^{post} points an outline to make them easier to see.

- At line 249, the authors indicate that orientation of "division places to preserve or avoid cell contacts" may be a more important aspect of pre-ACD BASL function. It seems to me the word "cell" is a bit too generic here... all the cells have contacts regardless of their identity. Perhaps they would consider revising it to something more specific such as to "avoid stomatal (or guard cell) contact" or "avoid contact between stomata".

We changed to … Orienting division planes to avoid the creation of stomata in contact

References cited in response to reviewers:
Dong, J., MacAlister, C. A., & Bergmann, D. C. (2009). BASL controls asymmetric cell division in Arabidopsis.
Cell, *137*(7), 1320-1330. https://doi.org/10.1016/j.cell.2009.04.018
Rowe, M. H., Dong, J., Weimer, A. K., & Bergmann, D. C. (2019). A Plant-Specific Polarity Module Establishes Cell Fate Asymmetry in the Arabidopsis Stomatal Lineage. *bioRxiv*, 614636. https://doi.org/10.1101/614636 Simmons, A. R., Davies, K. A., Wang, W., Liu, Z., & Bergmann, D. C. (2019). SOL1 and SOL2 regulate fate transition and cell divisions in the Arabidopsis stomatal lineage. *Development*, *146*(3). https://doi.org/10.1242/dev.171066

© 2021. Published by The Company of Biologists under the terms of the Creative Commons Attribution License (https://creativecommons.org/licenses/by/4.0/).

Second decision letter

MS ID#: DEVELOP/2021/199919

MS TITLE: Arabidopsis stomatal polarity protein BASL mediates distinct processes before and after cell division to coordinate cell size and fate asymmetries

AUTHORS: Yan Gong, Julien Alassimone, Andrew Muroyama, Gabriel Amador, Rachel Varnau, Ao Liu, and Dominique Bergmann
ARTICLE TYPE: Research Article

I am happy to tell you that your manuscript has been accepted for publication in Development, pending our standard ethics checks.

© 2021. Published by The Company of Biologists under the terms of the Creative Commons Attribution License (https://creativecommons.org/licenses/by/4.0/).
